# Cone-Shell Quantum Structures in Electric and Magnetic Fields as Switchable Traps for Photoexcited Charge Carriers

**DOI:** 10.3390/nano13101696

**Published:** 2023-05-22

**Authors:** Christian Heyn, Leonardo Ranasinghe, Ahmed Alshaikh, Carlos A. Duque

**Affiliations:** 1 Center for Hybrid Nanostructures (CHyN), University of Hamburg, Luruper Chaussee 149, 22761 Hamburg, Germany; leo92ra@outlook.com (L.R.); ahmed.alshaikh@uni-hamburg.de (A.A.); 2 Grupo de Materia Condensada-UdeA, Instituto de Física, Facultad de Ciencias Exactas y Naturales, Universidad de Antioquia UdeA, Calle 70 No. 52-21, Medellín AA 1226, Colombia; cduque_echeverri@yahoo.es

**Keywords:** semiconductor quantum ring, electric field, magnetic field, Aharonov-Bohm oscillations, trapping of photoexcited charge carriers, simulations

## Abstract

The optical emission of cone-shell quantum structures (CSQS) under vertical electric (*F*) and magnetic (*B*) fields is studied by means of simulations. A CSQS has a unique shape, where an electric field induces the transformation of the hole probability density from a disk into a quantum-ring with a tunable radius. The present study addresses the influence of an additional magnetic field. A common description for the influence of a *B*-field on charge carriers confined in a quantum dot is the Fock-Darwin model, which introduces the angular momentum quantum number *l* to describe the splitting of the energy levels. For a CSQS with the hole in the quantum ring state, the present simulations demonstrate a *B*-dependence of the hole energy which substantially deviates from the prediction of the Fock-Darwin model. In particular, the energy of exited states with a hole $l_{h}>$ 0 can become lower than the ground state energy with $l_{h}=$ 0. Because for the lowest-energy state the electron $l_{e}$ is always zero, states with $l_{h}>$ 0 are optically dark due to selection rules. This allows switching from a bright state ($l_{h}=$ 0) to a dark state ($l_{h}>$ 0) or vice versa by changing the strength of the *F* or *B* field. This effect can be very interesting for trapping photoexcited charge carriers for a desired time. Furthermore, the influence of the CSQS shape on the fields required for the bright to dark state transition is investigated.

## 1. Introduction

The quantized energy levels of charge carriers confined in a semiconductor quantum dot (QD) are split in a magnetic field [1,2,3,4,5,6] in a way analogous to the Zeeman-splitting of atomic spectral lines. A common description for the QD energy states in a magnetic field is the Fock-Darwin model [7]. There, a flat QD is considered in a radially-symmetric parabolic potential $V=\frac{1}{2}m^{*}\omega_{0}^{2}r^{2}$ with the effective mass $m^{*}$ of the charge carrier, the oscillator frequency $\omega_{0}$, and the radial position *r*. The assumption of a flat QD reflects the shape of common types of self-assembled QDs [8,9,10,11,12,13,14], where the elongation along the *z*-direction is significantly smaller compared to the x,y-directions. Accordingly, the *z*-quantization is always in the ground state and the level separation is caused by the x,y-quantization. The eigenenergies of the resulting two-dimensional harmonic oscillator are provided by the Fock-Darwin model [7]:

(1) $$E_{n,l}=\left(2n+|l|+1\right)\hbar {\left(\omega_{0}^{2}+\frac{1}{4}\omega_{c}^{2}\right)}^{\frac{1}{2}}\pm \frac{1}{2}l\hbar \omega_{c},$$

with the radial quantum number *n* = 0, 1, 2,..., the angular momentum quantum number *l* = 0, ±1, ±2,..., the cyclotron frequency $\omega_{c}=eB/m^{*}$, the absolute value of the elementary charge *e*, the magnetic strength *B*, and the last term being added for electrons and subtracted for holes.

Semiconductor QDs provide a confinement for electrostatically coupled electrons and holes, the so-called excitons. This means that the *B*-field dependence of the optical emission is controlled by the angular momentum quantum numbers of both the electron $l_{e}$ and the hole $l_{h}$ (we assume in the following the case that *n* = 0), where selection rules require for optical transitions that $l_{e}=l_{h}$.

**Figure 1:**
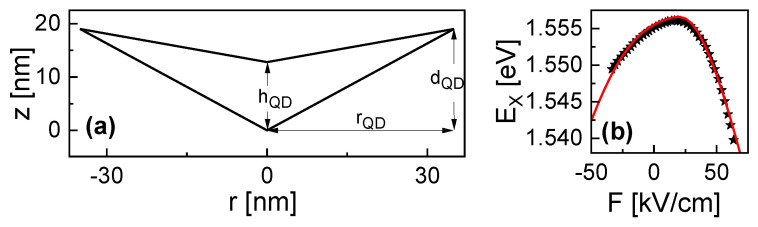
(**a**) Cross-section of a studied GaAs CSQS which is embedded in AlGaAs with indicated structural parameters and equal scales of the *r* and *z*-axis. The *F* and *B* fields are applied along the *z*-direction. (**b**) Measured (symbols) and simulated (line) *F*-dependent shift of the neutral exciton energy $E_{X}$ (Stark-shift) for the CSQS from (**a**). The experimental data are taken from [14].

The present study describes numerical simulations of the energy states in cone-shell quantum structures (CSQS) under vertical (along the *z*-direction in Figure 1a) electric (*F*) and magnetic (*B*) fields. CSQSs are fabricated using the self-assembled local droplet etching [15] technique during molecular beam epitaxy. As a peculiarity, CSQSs have a unique shape in which a vertical electric field allows shifting the wave functions (WF) along the surface of a cone. Figure 1a shows a cross-section of a CSQS. The approximated shape is assumed to be rotational symmetric. A previous study [14] on the influence of a vertical electric field demonstrated a large charge carrier separation, which yields an asymmetric and very strong quantum-confined Stark effect (Figure 1b) and a transformation of the hole WF from a disk into a ring-like shape. In the following, we focus on the behavior under an increasing positive electric field, where the hole WF transforms from disk-like into a ring-like shape. We note that due to the larger hole effective mass, the modification of the hole WF is more pronounced as compared to the according modification of the electron WF at negative electric fields. Regarding the influence of an additional magnetic field, in the disk shape, the electron and hole WFs follow approximately the prediction of the Fock-Darwin model; however, considering the case of a positive electric field F>0, the simulations indicate a substantially different behavior for the holes with a finite angular momentum of the ground state, which incrementally increases with the magnetic field. Here, a hybrid state is formed in which the hole has a ring-shaped WF and the electron has a zero momentum disk ground state. Now, optical selections rules allow switching from bright to dark states by changing the strength of the *F* or *B* fields.

## 2. Simulation Model

An electron and a hole confined in a quantum structure form an exciton (X), the energy of which can be described by the Hamiltonian

(2) $$H_{X}=H_{e}+H_{h}-H_{C},$$

with the electron and hole single-particle Hamiltonians $H_{e}$, $H_{h}$ and the Hamiltonian $H_{C}$ describing the attractive Coulomb interaction between both. The Coulomb interaction energy is described by a corresponding Hamiltonian in Cartesian coordinates:

(3) $$H_{C}=\frac{e^{2}}{4\pi \epsilon \epsilon_{0}\left|\vec{r_{e}}-\vec{r_{h}}\right|},$$

with the semiconductor permittivity ϵ, the vacuum permittivity $\epsilon_{0}$, and the electron and hole position vectors $\vec{r_{e}}$, $\vec{r_{h}}$.

In the effective mass approximation and assuming cylindrical symmetry, the single particle Hamiltonian in vertical electric *F* and magnetic fields *B* becomes [6]

(4) $$H_{j}=\frac{1}{2m_{j}^{*}}{\left(i\hbar \vec{\nabla_{j}}+q_{j}\vec{A_{j}}\right)}^{2}-q_{j}F\left(z-z_{0}\right)+V_{j}\left(\vec{r}\right),$$

where j=e for electrons and j=h for holes (the associated charge is $q_{e}=-e$ and $q_{h}=+e$), $m_{j}^{*}$ is the effective mass, *ℏ* is Planck’s reduced constant, $\vec{\nabla_{j}}$ is the gradient operator, $\vec{A_{j}}=-\frac{1}{2}\vec{r_{j}}\times \vec{B}$ is the vector potential used to describe *B*, *z* is the position along the direction of the electric and magnetic field, $z_{0}$ is the zero point of the electric field, and $V_{j}\left(\vec{r}\right)$ is the confinement potential. We assume here that the coordinates *r* and *z* related to the material induced potential *V* and to the electric field *F* do not depend on the charge carrier type. On the other side, due to a possible interaction in a magnetic field, the electron and orbital moments are probably not conserved separately and a charge carrier type dependent $r_{j}$ is considered in $A_{j}$. Adopting the Coulomb gauge condition, $\vec{A_{j}}$ must satisfy $\vec{\nabla_{j}}\cdot \vec{A_{j}}=0$. Now, Equation (4) becomes

(5) $$H_{j}=-\frac{\hbar^{2}}{2m_{j}^{*}}\nabla_{j}^{2}+\frac{i\hbar q_{j}}{m_{j}^{*}}\vec{A_{j}}\cdot \vec{\nabla_{j}}+\frac{q_{j}^{2}}{2m_{j}^{*}}A_{j}^{2}-q_{j}F\left(z-z_{0}\right)+V_{j}\left(\vec{r}\right).$$

In the present study, the magnetic field is always applied in the growth direction *z*, hence, $\vec{B}$ = (0, 0, *B*). Rearranging Equation (5) with $\vec{r}=x\hat{x}+y\hat{y}+z\hat{z}$ ($\hat{x},\hat{y},\hat{z}$ are unit vectors) yields

(6) $$H_{j}=-\frac{\hbar^{2}}{2m_{j}^{*}}\nabla_{j}^{2}+\frac{i\hbar q_{j}B}{2m_{j}^{*}}\left(y\frac{\partial }{\partial x}-x\frac{\partial }{\partial y}\right)+\frac{q_{j}^{2}B^{2}}{8m_{j}^{*}}\left(x^{2}+y^{2}\right)-q_{j}F\left(z-z_{0}\right)+V_{j}\left(\vec{r}\right).$$

With the momentum operator $p=-i\hbar \vec{\nabla }$ and the orbital angular momentum operator $L=\left(L_{x},L_{y},L_{z}\right)=-i\hbar \left(\vec{r}\times \vec{\nabla }\right)$, Equation (6) can be rewritten in terms of operators as follows:

(7) $$H_{j}=\frac{p_{j}^{2}}{2m_{j}^{*}}+\frac{q_{j}BL_{z}}{2m_{j}^{*}}+\frac{q_{j}^{2}B^{2}}{8m_{j}^{*}}\left(x^{2}+y^{2}\right)-q_{j}F\left(z-z_{0}\right)+V_{j}\left(\vec{r}\right).$$

The solutions of the Schrödinger equation with the Hamiltonian of Equation (7) are calculated numerically using the finite element method with the partial differential equation solver of the software COMSOL Multiphysics 6.0 (www.comsol.com). For this,

(8) $$c_{j}{\left(\frac{l_{j}}{r}\right)}^{2}-\frac{\hbar q_{j}Bl_{j}}{2m_{j}^{*}}+\frac{q_{j}^{2}B^{2}r^{2}}{8m_{j}^{*}}-q_{j}F\left(z-z_{0}\right)+V_{j}\left(r,z\right)$$

is used as input for the COMSOL solver, with the constant $c_{j}$ and the angular momentum quantum number $l_{j}$ (eigenvalue of the operator $L_{z}$). The width and height of the simulation field are $6r_{QD}$ and $3d_{QD}$ (see Figure 1a), respectively.

Details about the consideration of the material controlled potential $V_{j}\left(\vec{r}\right)$ are provided in a previous publication [14]. After computing the electron and hole wave functions inside the CSQS and the corresponding eigenenergies $E_{e}$, $E_{h}$, the Coulomb interaction energy $C_{eh}$ is calculated from the WFs via the Coulomb integral. Now, the neutral exciton energy is taken from

(9) $$E_{X}=E_{g}+E_{e}+E_{h}-C_{eh},$$

with the band-gap energy $E_{g}$ of the GaAs CSQS material. For the following simulations, a temperature of *T* = 4 K is assumed. Experimentally, the exciton energy is measured using micro-photoluminescence spectroscopy at liquid helium temperature [16].

We start the simulations with a rotational-symmetric CSQS shape approximation (Figure 1a), which has been introduced in a previous publication [14]. We note that in this model, for the sake of simplicity we neglect the self-consistency of the solution, in particular, breakage of rotational symmetry due to the Coulomb interaction. Nevertheless, the accuracy of the simulation model using this approximation is demonstrated by the very good reproduction of experimental neutral exciton Stark-shift data from a corresponding CSQS sample by model results (Figure 1b). Further shapes are addressed in Section 3.3.

## 3. Results and Discussion

The present simulations assume that in the ground state the radial quantum number is always *n* = 0. Varied parameters are the strengths of the electric *F* and magnetic *B* fields as well as the electron and hole angular momentum quantum numbers $l_{e}$, $l_{h}$.

### 3.1. Single-Particle Frame

**Figure 2:**
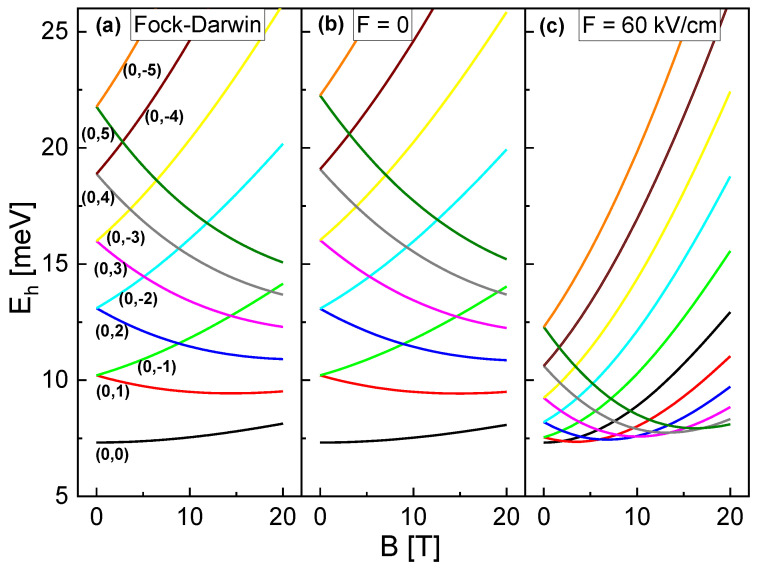
Calculated hole energy $E_{h}$ of the CSQS from Figure 1a as function of *B*. The line colors indicate the respective electron and hole angular momentum quantum numbers ($l_{e}$,$l_{h}$); the radial quantum number *n* is always zero. (**a**) Calculations using the Fock-Darwin model. For a better comparison with the simulations, $\omega_{0}$ is chosen according to the simulated quantization energy $E_{h}\left(B=0,l_{h}=1\right)-E_{h}\left(B=0,l_{h}=0\right)$ along the *r*-direction and the values are offset by the quantization energy $E_{h}\left(B=0,l_{h}=0\right)$ along the *z*-direction. (**b**) Simulated $E_{h}$ at *F* = 0. (**c**) Simulated $E_{h}$ at *F* = 60 kV/cm. For a better comparison, the zero point $z_{0}$ of the electric field is selected such that $E_{h}(B=0,F=60$ kV/cm$)=E_{h}\left(B=0,F=0\right)$.

As a first step, simulations are performed addressing the influence of combined *F* and *B* fields on the energy of the single-particle electron and hole states in the CSQS of Figure 1a. Figure 2a,b compares the hole energy $E_{h}$ for *F* = 0 at varied *B* and $l_{h}$ as calculated using the Fock-Darwin model and and numerical simulation. The behavior is well established, and for B=0 shows a constant offset with increasing $|l_{h}|$. An increase of *B* causes an increasing $E_{h}$ for $l_{h}\leq 0$ and a reduction of $E_{h}$ for $l_{h}>0$. We note that $E_{e}$ follows the same trend for a variation of *B* and $l_{e}$, however, with opposite behavior for $l_{e}$ being positive or negative and with different absolute values due to the smaller electron effective mass and consequently the larger $\omega_{0}$ and $\omega_{c}$.

For *F* = 0, a comparison with simulation results establishes a very good agreement of the simulated $E_{h}\left(B,l_{h}\right)$ (Figure 2b) and of $E_{e}\left(B,l_{e}\right)$ with the prediction of the Fock-Darwin model. In contrast, for *F* = 60 kV/cm the simulated $E_{h}$ clearly deviates from the predictions of the Fock-Darwin model (Figure 2c). Now, the level spacing is reduced and the *B*-dependence is much stronger. This behavior of $E_{h}$ represents the central finding of this section and is related to a transformation of the hole wave function shape inside the CSQS from a disk at F=0 into a ring at *F* = 60 kV/cm. On the other side, the simulated electron energy $E_{e}\left(B,l_{e}\right)$ for *F* = 60 kV/cm continues to agree well with the prediction of the Fock-Darwin model.

**Figure 3:**
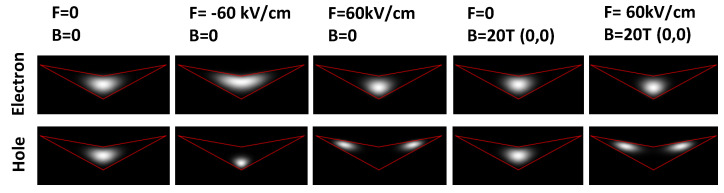
Gray scale plots of cross sections through simulated electron and hole probability densities inside the CSQS of Figure 1a at different vertical electric *F* and magnetic *B* fields. The angular momentum quantum numbers ($l_{e}$, $l_{h}$) are always zero. The red lines indicate the shape of the CSQS.

To illustrate the different regimes, Figure 3 shows simulated electron and hole probability densities $\psi_{e}^{2}$, $\psi_{h}^{2}$ under various *F* and *B* fields. At F=0 and B=0, both electron and hole have an almost disk-like shape. A negative *F* = −60 kV/cm yields only a slight lateral widening of the electron, and modifies the hole into a more sphere-like shape. At a positive *F* = 60 kV/cm the electron becomes more sphere-like, whereas the hole is fundamentally modified and transformed into a distinct ring shape. This different behavior of the electron and hole is related to their respective effective masses. Electrons with smaller effective mass have a higher probability density in the barrier material compared to the heavy holes. As a consequence, holes are more effectively squeezed into the shape of the structure, and can be shifted more easily into the wing (top) part, where they form a ring with *F*-dependent radius [14]. An additional magnetic field causes only a weak modification of the sphere and disk-like WF shapes. For the ring-shaped hole, a slight reduction of the radius is observed (*F* = −60 kV/cm, *B* = 20 T). 

To summarize the single-particle results, two regimes can be identified regarding the shape of charge carrier WFs. Disk-shaped and sphere-shaped charge carriers agree with the prediction of the Fock-Darwin model, where the ground-state ($l_{e}$ = 0, $l_{h}$ = 0) is always the state with the lowest energy. On the other side, the ground state of the ring shaped WF behaves differently. As a consequence of the reduced level spacing and the stronger *B*-dependence, the energy of states with $l_{h}>0$ can drop below the energy of the $l_{h}=0$ state at an elevated *B* (Figure 2c). For the example shown in Figure 2c, the respective state with the ground state $l_{h}$ increases by one at *B* = 2.1 T, 6.1 T, 9.8 T, 13.5 T, and 17.1 T, respectively. This behavior is relevant for the optical emission, which is discussed in the following section.

### 3.2. Multi-Particle Frame

**Figure 4:**
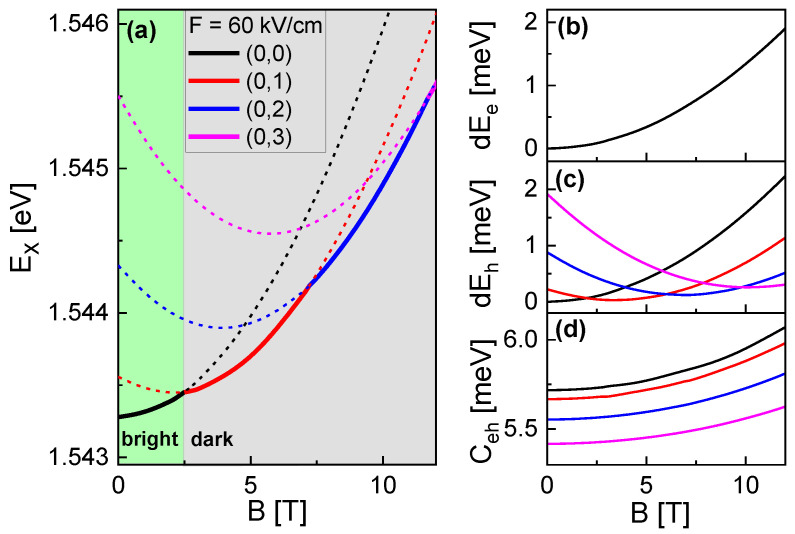
(**a**) *B*-field dependent exciton energy $E_{X}$ at *F* = 60 kV/cm and indicated ($l_{e}$,$l_{h}$). Solid lines indicate the states $E_{X0}$ with the lowest energy, while states with higher energy are plotted using dashed lines. The transition from a bright state (green area) with $l_{h}$ = 0 into a dark state (gray area) with $l_{h}\geq$ 1 takes place at *B* = 2.48 T. (**b**) *B*-dependence of the relative electron energy $dE_{e}=E_{e}\left(B\right)-E_{e}\left(B=0,l_{e}=0\right)$. The data for varied $l_{h}$ are identical. (**c**) *B*-dependence of the relative hole energy $dE_{h}=E_{h}\left(B\right)-E_{h}\left(B=0,l_{h}=0\right)$. The color code for the varied $l_{h}$ is provided in (**a**). (**d**) *B*-dependence of the Coulomb interaction energy $C_{eh}$. The color code for the varied $l_{h}$ is provided in (**a**).

Figure 4a shows simulated values of the exciton energy $E_{X}=E_{g}+E_{e}+E_{h}-C_{eh}$ at *F* = 60 kV/cm, $l_{e}$ = 0, and varied *B* and $l_{h}$. As the most important point, at higher *B*, states with $l_{h}>$ 0 can become the lowest-energy state $E_{X0}$. In Figure 4b–d, the *B*- and $l_{h}$-dependence of the corresponding contributions $E_{e}$, $E_{h}$, and $C_{eh}$ are plotted. There, the value of $E_{e}$ is not influenced by $l_{h}$- and the *B*-dependences of $C_{eh}$ for different $l_{h}$ are almost parallel. This identifies $E_{h}$ as the central contribution which controls the $l_{h}$-dependence of $E_{X0}$ (see Section 3.1). Further simulations indicate that $E_{X0}$ always includes an electron with a zero $l_{e}$, which is in agreement with the Fock-Darwin model. Considering that optical transitions between electrons and holes require $l_{e}=l_{h}$, only states with $l_{h}$ = 0 can be optically bright. As a consequence, the transition at *B* = 2.48 T from $l_{h}$ = 0 to 1 is associated with an expected bright to dark transition. For conditions with a low excitation power, where the CSQS is occupied only by one exciton, the bright to dark transition is expected to provide a mechanism for a controlled storage of photoexcited carriers. 

As a further interesting observation, the *B*-dependence of the lowest-energy state $E_{X0}$ in Figure 4a shows an oscillatory behavior. Magnetic field-dependent oscillations of the energy levels of charge carriers confined in a nanometer-scale ring are well known as Aharonov–Bohm (AB) oscillations [17] and are caused by quantum-interference phenomena. However, AB oscillations are a property of charged particles, and they are not expected for neutral particles such as excitons. On the other side, for the present CSQSs, the strong *E*-field induced polarization provides a significant charge-carrier separation, and as such allows the excitonic oscillations of $E_{X0}$ in Figure 4a. Considering the above selection rule $l_{e}=l_{h}$, only the first oscillation period is expected to be visible. To overcome this limitation, in spectroscopy, more oscillation periods might become visible at conditions with a high excitation power where excited states with lower $l_{h}$ or $l_{e}>0$ are occupied as well.

### 3.3. Influence of the CSQS Shape

**Figure 5:**
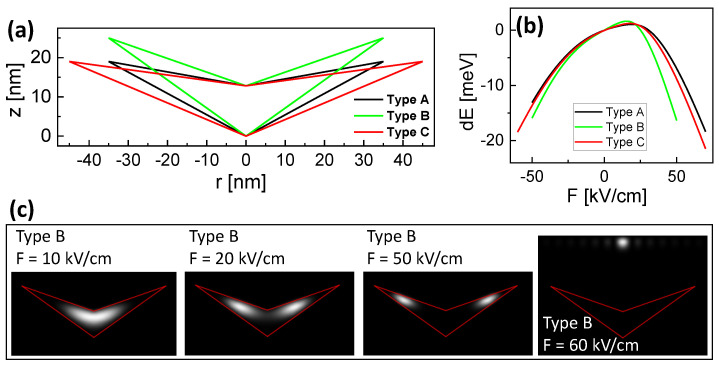
(**a**) Cross-sections of studied CSQSs with varied shape. The scales of the *r*-axis and *z*-axis are equal. (**b**) Simulated Stark shift at *B* = 0 for the different CSQS shapes. (**c**) Gray scale plots of cross-sections through the simulated hole probability densities of CSQS type B at varied *F*, *B* = 0, and ($l_{e}$,$l_{h}$) = (0,0). The red lines indicate the shape of the CSQS.

Thus far, we have studied the properties of a CSQS with a shape which is already experimentally realized and studied with microphotoluminescence spectroscopy under a varied *F*-field [14]. The shape is shown in Figure 1a and the corresponding Stark-shift data in Figure 1b, together with the respective simulation results. The relevant structural parameters are the CSQS radius $r_{QD}$ = 35 nm, the outer depth $d_{QD}$ = 19 nm, and the structure height $h_{QD}$ = 12.8 nm at the center (see Figure 1a). In the following, this shape is called type A. To evaluate the influence of the CSQS shape for future optimization of the fabrication scheme, we now study an additional shape with a deeper $d_{QD}$ = 25 nm called type B and a shape with a wider $r_{QD}$ = 45 nm called type C. The three types are compared in Figure 5a. Figure 5b shows the simulated Stark shifts for the different types. As expected, a larger size promotes a stronger charge-carrier separation and consequently a more pronounced slope of the Stark shift (types B and C). Here, the extension in *z*-direction is found to be decisive, as *F* points in this direction (type B). Furthermore, the simulations indicate that the range of useful values for *F* is limited. This is illustrated in Figure 5c, where the hole inside CSQS type B forms a disk at fields up tp 10 kV/cm, a ring between *F* = 20 and 50 kV/cm, and residues outside the structure for *F* = 60 kV/cm. The electric field-induced charge-carrier escape from a quantum dot is well known [18] and yields a limit for the maximum field. For the present CSQSs, the obtained useful ranges are plotted in Figure 5b, with type B up to *F* = 50 kV/cm and types A and C up to *F* = 60 kV/cm. Obviously, regarding the maximum possible Stark-shift, the limited field range of the type B structure compensates for the advantage of the stronger slope of the Stark shift.

**Figure 6:**
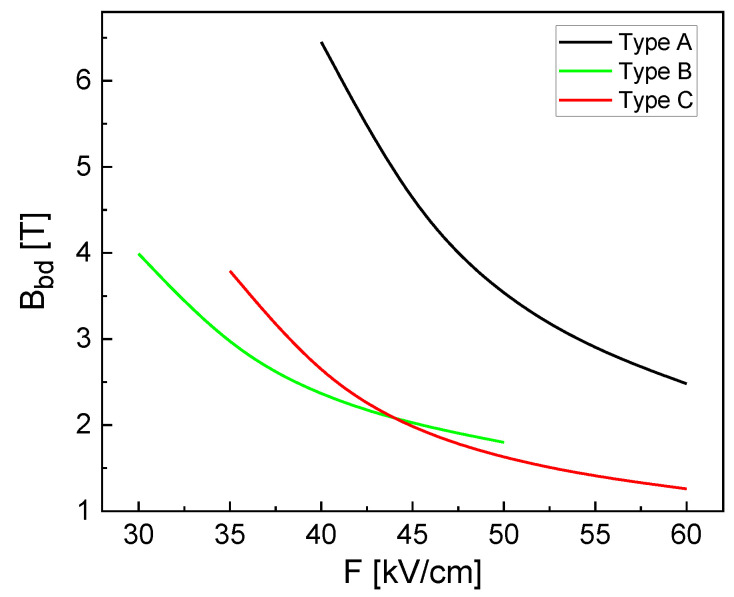
Magnetic field $B_{bd}$ at the transition from a bright state ($l_{h}=$ 0) into a dark state ($l_{h}\geq$ 1) as a function of *F* at varied CSQS shape.

In the next step, we simulate the magnetic field $B_{bd}$ at the transition from a bright state ($l_{h}=$ 0) into a dark state ($l_{h}\geq$ 1) as a function of *F* (Figure 6). If a high *F*-field is more easily achievable experimentally than a high *B*-field, the larger structures types B and C are advantageous. For type C at *F* = 60 kV/cm, the bright to dark transition takes place already at a low $B_{bd}$ = 1.26 T, which can be achieved by a low-cost permanent magnet. In the case of a constant *B*-field, the transition is switched by the *F*-field. As a huge advantage, this configuration would allow chip-based integration of a CSQS together with a permanent magnet to realize a switchable trap for photoexcited charge carriers.

## 4. Conclusions

The most intriguing aspect of the discussed cone-shell quantum structures is the charge-carrier configuration at an elevated electrical field, with a hybrid state composed of an electron confined as a quantum dot and a hole shaped like a tunable quantum ring (QR). QRs are a fascinating class of quantum structures with intriguing properties [19]. A prominent example are quantum-interference effects of charge carriers in the closed trajectory of a nanometer-scale ring, where a phase shift causes *B*-field dependent oscillatory behavior of the energy levels, i.e., the well-known Aharonov–Bohm oscillations [17]. In semiconductor QRs, AB-oscillations of charged particles are experimentally verified by means of transport [3], magnetization [20], or capacitance-voltage [21] measurements. However, because AB oscillations are a property of charged particles, they are expected to be only very weak for optical experiments dealing with neutral excitons.

To observe AB-oscillations optically, the excitons can be polarized using strain or electric fields. This is theoretically predicted [22,23,24,25] and experimentally demonstrated [26]. Here, the polarization causes different trajectories for electrons and holes, which yields an oscillating exciton ground-state energy. However, the optical AB-oscillations in structures with a type-I band alignment are only very weak. More pronounced AB-oscillations are observed when switching to structures using material systems with a type-II band alignment, such as InP/GaAs [27], ZnTe/ZnSe [28] or InAs/GaAsSb [29]. There, a strongly polarized hybrid state is realized, with one charge carrier forming a quantum dot and the other a quantum ring.

The present CSQSs provide such a hybrid dot-ring state for the strain-free GaAs/AlGaAs material system with a type-I band alignment, and the simulations predict clear AB-oscillations. However, due to the selection rule of equal electron and hole angular momentum quantum numbers, only the first period is expected to be bright. This allows for a controlled bright to dark transition by adjusting the strengths of the external *F* and *B* fields, and leads to the concept of a switchable trap for photoexcited charge carriers. For semiconductor QRs in a *B*-field, switching between bright and dark exciton ground states was theoretically predicted by Govorov et al. [23] and later by Fischer et al. [24], with both assuming an idealized ring shape. Here, we predict this effect for an already experimentally realized structure, where an electric field allows tuning between a dot and a hybrid dot-ring state. Using resonant excitation at the energy of the bright to dark transition, the exciting photon and the photon emitted from the CSQS should have the same energy after switching back from dark to bright. In a next step, magneto-photoluminescence measurements are desirable to verify the predicted trapping of photoexcited charge carriers and to evaluate possible storage times. 

## Data Availability

The data presented in this study are available on request from the corresponding author.
